# Impact of a Bundle of Interventions on the Spectrum of Parenteral Drug Preparation Errors in a Neonatal and Pediatric Intensive Care Unit

**DOI:** 10.3390/jcm13206053

**Published:** 2024-10-11

**Authors:** Sabine von Hobe, Mark Schoberer, Thorsten Orlikowsky, Julia Müller, Nina Kusch, Albrecht Eisert

**Affiliations:** 1 Hospital Pharmacy, RWTH Aachen University Hospital, 52074 Aachen, Germany; aeisert@ukaachen.de; 2 Section of Neonatology, Department of Pediatric and Adolescent Medicine, RWTH Aachen University Hospital, 52074 Aachen, Germany; mschoberer@ukaachen.de (M.S.); torlikowsky@ukaachen.de (T.O.); julmueller@ukaachen.de (J.M.); 3 Executive Department of Information Safety, RWTH Aachen University Hospital, 52074 Aachen, Germany; nikusch@ukaachen.de; 4 Institute of Clinical Pharmacology, RWTH Aachen University Hospital, 52074 Aachen, Germany

**Keywords:** neonatal intensive care, pediatric intensive care, medication errors, parenteral medications, drug preparation, interventional study, medication safety, error reduction, clinical pharmacist

## Abstract

**Background/Objectives**: This study aimed to evaluate the impact of a bundle of interventions on the error rates in preparing parenteral medications in a neonatal and pediatric intensive care unit (NICU/PICU). **Methods**: We conducted a prospective interventional study in a NICU/PICU in a tertiary university hospital as a follow-up to a prior study in the same setting. A clinical pharmacist and a pharmacy technician (PT) analyzed the workflow of drug preparation on the ward, identified high-alert medications, and defined a bundle of five interventions, which include the following: Drug Labeling: 1. EN ISO-DIVI labeling; Training: 2. Standardized preparation process on the ward; 3. eLearning Program; 4. Expert Consultations; and Location of Preparation: 5. Transfer of the preparation of high-alert medications and standardized preparations to the central pharmacy. After implementing the bundle of interventions, we observed the preparation process on the ward to evaluate if the implementation of the interventions had an impact on the quality of the drug preparation. **Results**: We observed 262 preparations in the NICU/PICU. Each single step of the preparation process was defined as an error opportunity. We defined seven error categories with an overall error opportunity of 1413. In total, we observed 11 errors (0.78%). The reduction in the overall error rate from 1.32% in the former study to 0.78% per preparation opportunity demonstrated that the implemented interventions were effective in enhancing medication safety. **Conclusions**: This study provides evidence that a bundle of interventions, including standardizing drug labeling, enhancing training, and centralizing the preparation of high-alert medications, can reduce medication errors in NICU/PICU settings.

## 1. Introduction

Medication errors (MEs) are a significant cause of adverse events in pediatric pharmacotherapy. In Pediatric Intensive Care Units (PICUs), MEs occur seven times more frequently than in other pediatric inpatient units [1]. Neonates are at particularly high risk for medication errors [2], with studies indicating error rates ranging from 13 to 91 per 100 admissions in NICUs. The error rate is higher in preterm compared to term-born neonates (75.4% vs. 24.6%) [3].

Many drugs used in pediatric ICUs are approved only for adults and are prescribed off-label. There is a lack of age-appropriate dosage forms, and sufficient dosage or preparation information is often not available. Since medications in pediatric ICUs are mainly administered parenterally, multiple manipulation steps are required in the drug preparation process [4]. Due to the variability in organ maturity and therefore age-related changes in pharmacokinetic and pharmacodynamic parameters, dosages must be individually calculated based on body weight or surface area, increasing the risk of MEs [5,6]. Moreover, neonates and critically ill pediatric patients often require highly concentrated doses and smaller volumes of medication, leading to a higher probability of dosing miscalculation and dilution errors. The preparation of parenteral medication is complex, involving nursing staff at the bedside, in a preparation room on the ward, pharmacy technicians (PT) in a satellite pharmacy, or in the pharmacy department’s clean room [7]. The impact of MEs is often more severe, as a large proportion of NICU/PICU medications are classified as high-alert medications [8,9].

Medication preparation on the ward is typically performed by nursing staff. Due to limited resources and time constraints, this task is often carried out by a single person, increasing the likelihood of errors that are not detected before reaching the patient. A systematic review of a Critical Incident Reporting System (CIRS) registry found that 44% of errors occurred during drug preparation [10].

Various interventions have been shown to reduce drug preparation errors. A systematic review by Koeck et al. regarding the quality improvement of preparation and administration in NICU/PICU settings found that “administrative controls”, such as preparation guidelines or education aimed at behavioral changes in personnel, significantly reduced error rates [11]. The interventions included implementing standardized dilutions, a pharmacist production unit, standardized labels, standardized preparation lists, education and/or practical training programs, eLearning programs, and expert consultations conducted by a PT. In four of the reviewed studies only a single intervention [12,13,14,15], in two studies a bundle of interventions was implemented [3,16]. Koeck et al. concluded that a combination of interventions in NICU/PICU settings was more effective than a single intervention, although the impact of each intervention could not be separately assessed [11].

Niemann et al. (2014) could reduce the error rate of at least one medication in drug handling from 88% to 49% in a three-step intervention in the form of a handout, a training course, and a reference book [12]. Another study with the intervention of a pharmacist-led presentation including the errors of the preintervention phase for the nursing staff reduced the errors from 44.3% to 28.6% [17]. Chedoe et al. decreased the incidence of errors from 49% to 31% by implementing theoretical and practical training for nurses in a NICU [13].

Another recommended intervention includes preparing critical parenteral medications in a central pharmacy [7,18,19].

In a prior study conducted in the NICU/PICU of University Hospital RWTH Aachen, Hermanspann et al. analyzed MEs during parenteral drug preparation [20]. This follow-up study aimed to implement a bundle of interventions and evaluate whether error rates in the medication preparation process can be reduced compared to the previous study by Hermanspann et al., thereby enhancing medication safety.

## 2. Materials and Methods

We conducted an interventional study in the NICU/PICU of University Hospital RWTH Aachen. This study has been approved by the local ethics committee (EK 106/21) and by the local data protection officer of the medical faculty of RWTH Aachen University.

Based on the findings of errors in the preparation process by Hermanspann et al. [20], we analyzed the workflow on the ward to identify critical processes. Between March and November 2020, the clinical pharmacist and a pharmacy technician specialized in drug preparation (PT) together with the ward manager, responsible for the preparation process, representing all nursing staff, broke down the workflow from prescription to the preparation into individual steps to analyze them separately. NICU/PICU physicians prescribed the medication using the CPOE VISITE 2000, Version 3.3.5, Lyomark Pharma GmbH, Oberhaching, Germany. The ward manager explained how the preparation steps were performed. Then, the pharmacist and PT observed the procedures performed by the nurses on weekdays during the drug preparation process.

The clinical pharmacist developed an intervention strategy consisting of five interventions based on literature and former examinations by Hermanspann et al. [20] and the assessment of the critical preparation steps.

Critical processes and the selection of the interventions were discussed openly with consent from two other trained clinical pharmacists, the senior physician, the head of NICU/PICU, and the ward manager.

The following five interventions in three categories were implemented (Table 1):

Drug Labeling: We implemented the exclusive use of standardized labels recommended by the German Interdisciplinary Association for Intensive Care and Emergency Medicine (DIVI), adapted to EN ISO 26825 [21]. The labels are specifically designed for each drug class. Additionally, the patient ID, name, and concentration of the active substance, dose [mg], and volume [mL] are printed on the label. From August 2021, we used the etiMed label printer by Etifix, Grafenberg, Germany. The nurses were trained to use the printer. A standard operating procedure (SOP) defined how each nurse had to label every prepared drug.

Training: We developed a checklist to standardize the preparation process on the ward and to enhance awareness of critical processes. Especially the correct implementation of the double check principle was in focus (Table S1).

The clinical pharmacist and a PT created an eLearning video in February 2021 in cooperation with the Department of Pediatric and Adolescent Medicine, Section of Neonatology, the Institute of Hygiene and Infectiology, and the communications department. The video was filmed inhouse with experienced personnel, featuring the various steps along the checklist. The key points were emphasized through additional text overlays. This intervention aimed at teaching the correct preparation technique to standardize the preparation process on the ward. Each nurse was obliged by the ward management to watch the video; the participation was centrally documented.

Additionally, between February and May 2021, we initiated a weekly expert consultation on the ward. The expert (PT) could be contacted at any time during the day regarding questions about preparation techniques. The PT trained the nurses directly on the ward by preparing dummy formulations and simulating the process according to the checklist.

To assess the effectiveness of the training, the nurses were asked to fill out a questionnaire consisting of 16 questions (Table S2). Among other questions, the nurses were asked to what extent they had gained knowledge about the preparation of parenteral drugs during their professional education, if they felt safe in preparing and administering parenteral medication, and if they considered the training a useful enhancement of their knowledge.

Location of preparation: We identified preparations with lipid ingredients as well as all parenteral nutrition preparations as high-alert medications due to their complex composition and high susceptibility to microbiologic contamination. Together with the senior physician, we defined standard parenteral nutrition admixtures, which could be prepared in advance in the central pharmacy and used in acute situations outside pharmacy operating hours. Also, we analyzed the type and number of drugs with a standardized dilution, which could be prepared in the pharmacy in predictable and not acute situations. Wherever possible, those preparations were transferred to the central pharmacy department.

Implementation of interventions and evaluation: Between February and September 2021, the interventions were implemented in the preparation process.

Between November 2021 and March 2022, the clinical pharmacist observed the nurses during the preparation of parenteral drugs to evaluate the impact of the interventions.

We observed a comparable number of preparations compared to those in the previous study by Hermanspann et al. [20]. Data collection was preceded by three pilot days so that the observer became familiar with the processes and medications on the ward. Drug preparations were observed from Monday to Friday in the daytime.

First, the observer introduced herself to each nurse and asked for permission for direct observation of the preparation of intravenous drugs. Only after verbal consent from the nurse did the observer start collecting data. The nurses were informed that an observation of the preparation was conducted, but they were not informed about the aim and different steps involved in this study, so the observation was disguised. For privacy reasons, neither patient data nor the names of the nurses were collected.

As this study was planned as a follow-up observation to Hermanspann et al. [20] we used the same study protocol in the same team and setting.

The data collection form was standardized according to the preparation process. The categories of medication errors contained the name of the medication, type, and volume of the carrier solution, process of mixing, reconstitution solvent if applicable, dose, and labeling.

We used the guidelines of the Society of Hospital Pharmacists to prevent ME [22]. We classified the preparation of the drug into the following: 1. liquid concentrate in a syringe; 2. liquid concentrate with diluent in a syringe or infusion bag; and 3. powdered medication to be reconstituted and dissolved, diluted if necessary. The observer only stopped the preparation process when she detected a serious preparation error that could harm the patient.

### 2.1. Definitions

The National Coordinating Council for Medication Error Reporting and Prevention (NCC MERP) defines a ME as “any preventable event that may cause or lead to inappropriate medication use or patient harm while the medication is in the control of the health care professional, patient, or consumer. Such events may be related to professional practice, health care products, procedures, and systems, including prescribing, order communication, product labeling, packaging, and nomenclature, compounding, dispensing, distribution, administration, education, monitoring, and use” [23].

The Institute for Safe Medications Practice (ISMP) defines drugs as high-alert medications that “bear a heightened risk of causing significant patient harm when they are used in error. Although mistakes may or may not be more common with these drugs, the consequences of an error are clearly more devastating to patients” [9].

As in the study by Hermanspann et al., we defined seven error categories that could occur in the preparation process: 1. Medication: drug preparation that differed from the physician’s prescription; 2. Type of solution: wrong diluent or solvent; 3. Volume of solution, wrong volume of solution or diluent that does not follow the package insert or local or national hospital guidelines or good manufacturing practice; 4. Uniform mixing: insufficient mixing of two solutions; 5. Full reconstitution: insufficient reconstitution of a powdered medication with the solvent; 6. Dose: dose that differed more than 5% from the physician’s prescription [20]; and 7. Labeling: incorrect labeling with missing information regarding medication name and concentration.

We did not evaluate the physician’s prescription or aspects of hygiene.

### 2.2. Data Analysis

Firstly, we calculated the overall error rate both as the percentage of errors occurring across the total number of observed preparations as well as the percentage of errors occurring across the total number of observed preparation steps, since every distinct preparation step presents an additional opportunity to commit an error. This was performed for both the data collected within the framework of this study and the data collected by Hermanspann et al. [20]. Subsequently, we used the Wilson score confidence interval in combination with Yate’s continuity correction to compute the 95% confidence intervals of the respective error rates. We specifically employed the Wilson score interval in order to address the particular challenges posed by the data sets collected in both studies due to its robustness, its accuracy, and its ability to handle highly skewed observations.

Additionally, we were interested in evaluating whether applying the interventions would result in a statistically significant decrease in said error rates. Since the sample sizes and both the total numbers of errors as well as correct preparations observed in both data sets are sufficiently large to meet the conditions of z-tests, we were able to perform one-tailed z-tests for proportions. We applied a level of significance of α=0.05.

Furthermore, the data of both studies were grouped with respect to the category of medication error that was observed. For each such category, we computed once again the total number of relevant preparations as well as the total number of observed errors, from which the respective error rate was calculated as a percentage. In addition, 95% confidence intervals for the error rates were calculated for each category. Due to low sample sizes and small proportions, we again chose the Wilson score confidence interval and applied Yate’s continuity correction. Lastly, due to the same limitations, Fisher’s exact test was utilized to compute *p*-values to evaluate whether the pairs of corresponding group-specific error rates reported by the two studies differ significantly. We applied a level of significance of α=0.05. In cases where no single error was observed within a specific group in both data sets, no confidence interval or test for statistical significance was computed. The objective of this study is to investigate the impact of a bundle of interventions on a spectrum of drug preparation errors: If a certain class of errors did not occur—neither before nor after implementing said bundle of interventions—we have no incentive to further investigate any possible impact on said class of errors.

All computations were performed using R (version 4.3.2) using prop.test, Binom.CI, and fisher.test.

## 3. Results

**Figure 1:**
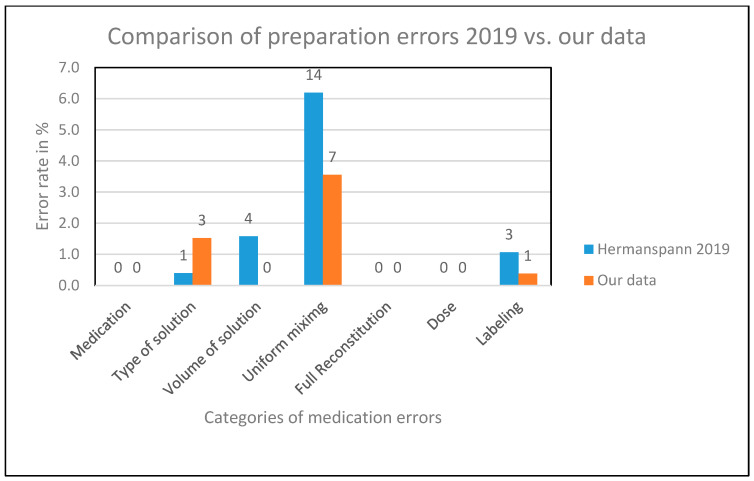
Comparison of preparation errors in 2019 [20] versus our data [% and absolute numbers].

In total, the clinical pharmacist observed 262 preparations. Since no safety-relevant errors occurred, interaction by the pharmacist was not necessary. The comparison of errors is summarized in an overview in Figure 1.

Within the framework of this study, we observed 11 errors across a total of 1413 opportunities, resulting in an overall error rate of 0.78% with a 95% confidence interval of [0.41%, 1.43%], as opposed to Hermanspann et al. [20], who reported 22 errors across 1671 opportunities with an overall error rate of 1.32% and a 95% confidence interval of [0.85%, 2.02%]. There was no case with more than one error in one preparation. With respect to the total number of 262 preparations, the 11 errors we observed produced an error rate of 4.17% (CI_95_ = [2.21%, 7.54%]), whereas Hermanspann et al. [20], reported an ME rate of 7.83% (CI_95_ = [5.08%, 11.78%]). After conducting statistical testing, we found weak statistical significance (*p* = 0.04) in the decrease in the error rate across all preparations by the standards of this study, that is, at a level of significance of 0.05. By contrast, we could not reject the null hypothesis regarding the decrease in the error rate reported across all error opportunities. The results are summed up in Table 2.

We note that certain data properties, in particular the small absolute number of errors—11 errors out of 1413 opportunities in this study]—and the highly skewed distribution of errors across categories, may restrict the reliability of the results of this statistical analysis, even though we took great care to apply sufficiently robust statistical methods. Conducting follow-up studies to investigate the reproducibility of these findings would be greatly beneficial.

After the implementation of the interventions, we identified fewer or no errors across all sub-categories with exactly one exception. In the category “type of solution”, we identified both an absolute as well as a relative increase in errors, producing an error rate of 1.52% in contrast to the error rate of 0.40% reported by Hermanspann et al. [20]. Subsequent statistical testing revealed that the null hypothesis regarding the significance of the observed difference in error rates could not be rejected. Therefore, this change in the error rate is not statistically significant and likely due to chance. In contrast, the error rates in the remaining three categories where at least one error was observed in either of the two studies decreased with respect to the rates reported by Hermanspann et al. [20]. The mixing error rate decreased from 6.19% to 3.55%, the labeling error rate was lowered from 1.07% to 0.38%, and the error rate associated with the volume of solution decreased from 1.58% to 0%. None of these differences in error rates was found to be statistically significant when conducting statistical testing. The results of the statistical analysis regarding the distinct categories of medication errors are presented in Table 3.

Results of the questionnaire (Table S2):

The nurses completed 31 out of 46 questionnaires. When asked how they rated the knowledge imparted during their professional education on the preparation of parenteral medications, 35.5% of the nurses answered with “just enough”, 32.2% with “rather insufficient”, and 3.2% with “insufficient”. Moreover, 48.4% of the nurses felt safe or very safe in preparing and administering parenteral medication, and 51.6% felt rather safe or less safe. 90.3% of the nurses considered the training as a useful enhancement of their knowledge.

## 4. Discussion

### 4.1. Key Results

The implementation of a bundle of interventions, including standardized labeling, training, and centralizing the preparation of high-risk medications, could reduce medication errors (MEs) in our study to an error rate of 0.78% per preparation opportunity, compared with data by Hermanspann et al. [20] with an error rate of 1.32%. In the study by Hermanspann et al. [20], the error rate in the medication preparation process on the ward was relatively low compared to other studies found in the literature. In this highly sensitive patient population, the reduction of errors in preparing parenteral medication by almost half is very meaningful, even though the absolute error rates are low.

Studies reported errors in intravenous drug preparations from 0 to 99% in different error types [7]. Hermanspann et al. [20] found an error rate of 1.32% for preparations on the NICU/PICU (and with administration: 1.5%).

In our study, errors occurred in the categories “type of solution (wrong solvent)”, “uniform mixing”, and “labeling”, but were reduced after implementation of the interventions.

The error rate in the category “uniform mixing of two solutions” (here defined as turning the vial or syringe at least three times) was reduced by 43% after implementation of the interventions. In literature, this category was also found to be the main error in the category “preparation errors” [13]. Although there are different definitions in the literature, Cousins analyzed 79% of errors reported as “product not mixed” [19], Chedoe noted 9% preintervention and 4% postintervention errors in the NICU [13].

As in the study of Hermanspann et al. [20], we observed no errors in the categories “medication”, “reconstitution”, and “dose”.

We found more errors in the category “type of solution” in this study than in 2019 [20], which was statistically not significant.

We evaluated the effectiveness of each specific intervention.

Standardized Drug Labeling: Implementing EN ISO-DIVI labeling contributed to the reduction in labeling errors from 1.07% to 0.38%. National and international organizations have recommended standardized colored syringe drug labeling Standard ISO 26825 for years [24,25]. These labels were already used in the NICU/PICU during the first observational period described by Hermanspann et al. [20], but often incompletely filled in. The one “label error” we observed occurred because an incorrect concentration was added manually. Standard operating procedures and training can enhance the correct use of standardized labeling even in stressful situations on the ward.

Training Programs: The introduction of an eLearning program, expert consultations, and a standardized preparation process checklist aimed to enhance the nurses’ competency and confidence in drug preparation. Other studies showed an error reduction by implementing training programs [12,13]. The positive feedback from the nurses, with 90.3% considering the training beneficial, underscores the importance of continuous education in reducing errors. The eLearning video was mainly valued as a useful extension of their knowledge (Table S2). The reported error rate in the mixing category decreased from 6.19% to 3.55%, indicating that training directly impacts the accuracy of complex preparation tasks.

Centralizing High-Risk Drug Preparation: Transferring the preparation of lipid-based and parenteral nutrition drugs as well as other high-alert medication preparations to the central pharmacy was implemented to minimize errors and contamination risks. Kaufmann et al. noted that pre-prepared and labeled syringes, commercially acquired or prepared in the hospital pharmacy, are more precise because of the preparation under controlled conditions in clean-room facilities and with established quality controls [24]. Although the specific impact on microbiological contamination was not assessed, the overall reduction in error rates supports the effectiveness of this strategy. Studies pointed out that the work environment has an impact on patient safety [26] and error reporting [27]. The number of patients per nurse can also influence the occurrence of ME [28]. Di Simone assumed that interruption of the preparation process and noise were possible reasons for medication errors [29].

Another advantage of shifting the preparation of high-alert medications to the central pharmacy is that drug therapy safety checks can identify additional potential risks. This is especially important for high-alert medications, as demonstrated in the study by Kiesel et al. regarding drug interactions [30].

An additional quality-enhancing factor is that qualitative and quantitative measurements can be conducted in the central pharmacy [19].

### 4.2. Strengths and Limitations

The strength of this study is that, to our knowledge, it is the first to analyze errors in the preparation of parenteral drugs, the implementation of a bundle of interventions, and its effect on error rates in the same setting, protocol, and design. This was an advantage because other studies are performed in different settings with different methods, using different error definitions, so it is difficult to compare the results. Additionally, it was evaluated whether the interventions provided support to the nursing staff during the process of preparing parenteral medications.

As in many other studies, there was only one trained observer, so we could not exclude an observer bias [31]. As we did not observe the preparation process during weekends or during night shifts, we did not assess the impact of the time of day on the errors. We did not observe the administration of parenteral medication. The clinical relevance of the errors was not discussed [13].

The nurses were informed that their preparation process would be observed, but they were not made aware of the specific aim of this study. Still, they could alter their behavior while being observed (Hawthorne effect). This could influence the results, although studies showed that the effect is not significant [32,33,34].

## 5. Conclusions

To our knowledge, this study is the first to evaluate errors in parenteral medication preparation within the same NICU/PICU setting. The findings demonstrate that a combination of interventions—such as standardizing drug labeling, enhancing training, and centralizing the preparation of high-alert medications—can significantly improve the safety and quality of pediatric pharmacotherapy.

## Figures and Tables

**Table 1:** Bundle of interventions.

Drug Labeling	1. EN ISO-DIVI labeling (standardized labels) [21]
Training	2. Standardized preparation process for the ward
	3. ELearning program
	4. Expert consultations (PT)
Location of Preparation	5. List of drugs of standardized dilutions that can be prepared in the pharmacy in predictable and not acute situations

**Table 2:** Overview of the workflow and results.

	Hermanspann et al. (2019) [20]	Our Data
Number of beds	18	14
Nurse-to-patient ratio	1.4	1.5
Observation period	09.2012–06.2013	11.2021–03.2022
Absolute number of errors	22	11
Absolute number of preparations observed	281	262
Absolute number of opportunities for errors	1671	1413
Error rate across observed preparations	r_err_ = 7.83%	r_err_ = 4.20%
	CI_95_ = [5.08%, 11.78%]	CI_95_ = [2.21%, 7.54%]
*p*-value: error rate across preparations	*p* = 0.04	
Error rate across observed error opportunities	r_err_ = 1.32%	r_err_ = 0.78%
	CI_95_ = [0.85%, 2.02%]	CI_95_ = [0.41%, 1.43%]
*p*-value: error rate across error opportunities	*p* = 0.07	

**Table 3:** Results for sub-categories of errors.

	Hermanspann et al. (2019) [20]		Our Data		
Category of Medication Error	Absolute Number of Preparations and Errors	Error Rate and 95% Confidence Interval	Absolute Number of Preparations and Errors	Error Rate and 95% Confidence Interval	*p*-Value: Differences between Error Rates
Medication	N_prep_ = 281	r_err_ = 0%	N_prep_ = 262	r_err_ = 0%	N/A
	N_err_ = 0	CI_95_ was not computed	N_err_ = 0	CI_95_ was not computed	
Type of solution	N_prep_ = 253	r_err_ = 0.40%	N_prep_ = 197	r_err_ = 1.52%	*p* = 0.32
	N_err_ = 1	CI_95_ = [0.02%, 2.53%]	N_err_ = 3	CI_95_ = [0.39%, 4.75%]	
Volume of solution	N_prep_ = 253	r_err_ = 1.58%	N_prep_ = 197	r_err_ = 0%	*p* = 0.14
	N_err_ = 4	CI_95_ = [0.51%, 4.27%]	N_err_ = 0	CI_95_ = [0.00%, 2.38%]	
Uniform mixing	N_prep_ = 226	r_err_ = 6.19%	N_prep_ = 197	r_err_ = 3.55%	*p* = 0.26
	N_err_ = 14	CI_95_ = [3.56%, 10.39%]	N_err_ = 7	CI_95_ = [1.57%, 7.48%]	
Reconstituted	N_prep_ = 96	r_err_ = 0%	N_prep_ = 36	r_err_ = 0%	N/A
	N_err_ = 0	CI_95_ was not computed	N_err_ = 0	CI_95_ was not computed	
Dose	N_prep_ = 281	r_err_ = 0%	N_prep_ = 262	r_err_ = 0%	N/A
	N_err_ = 0	CI_95_ was not computed	N_err_ = 0	CI_95_ was not computed	
Labeling	N_prep_ = 281	r_err_ = 1.07%	N_prep_ = 262	r_err_ = 0.38%	*p* = 0.62
	N_err_ = 3	CI_95_ = [0.28%, 3.35%]	N_err_ = 1	CI_95_ = [0.02%, 2.44%]	

## Data Availability

Data are available upon request from the corresponding author.

## Supplementary Materials

The following supporting information can be downloaded at: https://www.mdpi.com/article/10.3390/jcm13206053/s1. Table S1: Checklist for the preparation of parenteral medication; Table S2: Results of the questionnaire.
