# Supplementary material for: Impact of a Bundle of Interventions on the Spectrum of Parenteral Drug Preparation Errors in a Neonatal and Pediatric Intensive Care Unit
Source: J Clin Med. 2024 Oct 11;13(20):6053. doi: 10.3390/jcm13206053 (PMC11509000; doi:10.3390/jcm13206053)
Supplement: Supplementary file 1 [file jcm-13-06053-s001.zip › jcm-3229789-supplementary.pdf]

Journal of
*Clinical Medicine*

MDPI

**Table S1.** Checklist for the preparation of parenteral medication.

| | |
|---|---|
| Cleaning and disinfection of the preparation area | |
| **Preliminary**: | |
| **Drug-Check:** Verification and calculation with double check: | Right patient<br>Right drug<br>Right concentration<br>Right dose<br>Right solvent<br>Right volume of the solvent<br>Right diluent solution<br>Right volume of the diluent solution |
| **Label preparation** | Patient's name, name and quantity of the active ingredient, necessary volume, type, and volume of the diluent solution required |
| **Preparation** | 1. Preparation with the double-check principle<br>2. Verification of the type and quantity of the<br>♣ drug<br>♣ solvent<br>♣ diluent solution<br>3. Complete reconstitution when using powdered medication<br>4. Uniform mixing after dilution (defined as turning the vial or syringe at least three times according to our local hospital guidelines)<br>5. Freedom from particulate matter |

**Table S2.** Results of the questionnaire.

| | Questions | Answers |
|---|---|---|
| Question 1 | Are you a specialized nurse in intensive care medicine? | Yes: 15 (48.4%)<br>No: 15 (48.4%)<br>In further training: 1 (3.2%) |
| Question 2 | How long have you been working in the pediatric intensive care unit? | <1 y: 10 (32.2%)<br>1-5 y: 6 (19.4%)<br>>5 y: 15 (48.4%) |
| Question 3 | How do you rate to what extent you had gained knowledge about the preparation of parenteral drugs during your professional education or specialized further education? | Completely sufficient: 1 (3.2%)<br>Rather sufficient: 8 (25.8%)<br>Just enough: 11 (35.5%)<br>Rather insufficient: 10 (32.2%) Insufficient: 1 (3.2%) |
| Question 4 | How confident do you feel in the preparation and administration of parenteral medications based on your training and current level of information? | Very safe: 3 (9.7%)<br>Safe: 12 (38.7%)<br>Rather safe: 12 (38.7%)<br>Less safe: 4 (12.9%)<br>Unsafe: 0 |

| | | |
|---|---|---|
| Question 5 | Does the training program offer significant knowledge enhancement for you? | Fully applicable: 16 (51.6%) Mainly applicable: 12 (38.7%) Applicable: 3 (9.7%)<br>Rather not applicable: 0<br>Not applicable: 0 |
| Question 6 | In your opinion, does the training contribute to increased safety in parenteral medication preparation? | Fully applicable: 12 (38.7%) Mainly applicable: 12 (38.7%) Applicable: 6 (19.4%)<br>Rather not applicable: 0<br>Not applicable: 1 (3.2%) |
| Question 7 | Do you find the hygiene measures reasonable? | Yes: 30 (96.8%)<br>No: 0<br>Partially: 1 (3.2%) |
| Question 8 | Do you believe hygiene measures can be effectively implemented? | Yes: 16 (51.6%)<br>No: 0<br>Partially: 14 (45.2%)<br>NA: 1 (3.2%) |
| Question 9 | Do you find the demonstrated preparation steps reasonable? | Yes: 28 (90.3%)<br>No: 0<br>Partially: 2 (6.5%)<br>NA: 1 (3.2%) |
| Question 10 | Do you believe the demonstrated preparation steps can be effectively implemented? | Yes: 12 (38.7%)<br>No: 1 (3.2%)<br>Partially: 16 (51.6%)<br>NA: 2 (6.5%) |
| Question 11 | Do you find the demonstrated double-check procedure reasonable? | Yes: 29 (93.5%)<br>No: 0<br>Partially: 2 (6.5%) |
| Question 12 | Do you think the double-check procedure can be effectively implemented? | Yes: 18 (58.1%)<br>No: 1 (3.2%)<br>Partially: 12 (38.7%) |
| Question 11 | Do you find the labeling reasonable? | Yes: 29 (93.5%)<br>No: 0<br>Partially: 1 (3.2%)<br>NA: 1 (3.2%) |
| Question 12 | Do you think the labeling can be effectively implemented? | Yes: 24 (77.4%)<br>No: 1 (3.2%)<br>Partially: 3 (9.7%)<br>NA: 3 (9.7%) |
| Question 13 | Does implementing double checking enhance your sense of safety? | Fully applicable: 14 (45.2%)<br>Mainly applicable: 10 (32.3%) Applicable: 4 (12.8%)<br>Rather not applicable: 0<br>Not applicable: 1 (3.2%)<br>NA: 2 (6.5%) |

| | | |
|---|---|---|
| Question 14 | Which aspects of the workflow from prescription to application do you think can still be improved? | • Optimizing the workflow of prescriptions,<br>• Avoiding handwritten prescriptions,<br>• Implementing standard concentrations and detailed preparation instructions for each medication |
| Question 15 | Are there any other areas related to medication preparation that you would like to have more information on? | • Compatibility information when two or more drugs are mixed in one syringe |
| Question 16 | Any additional comments or suggestions for improvement? | • Positive feedback and appreciation of the support in enhancing the quality of preparation together with the nursing care staff |
